# Proliferative Diabetic Retinopathy in Young-Onset Type 1 Diabetes in Croatia: Risk Factors and a Predictive Economic Model for National Screening

**DOI:** 10.3390/medicina61122168

**Published:** 2025-12-05

**Authors:** Ivan Borjan, Ivna Pleština-Borjan, Ljubo Znaor, Maja Pavić, Tatjana Josifova, Irena Marković, Beáta Éva Petrovski, Goran Petrovski

**Affiliations:** 1 Department of Ophthalmology, University Hospital Split, Spinciceva 1, 21000 Split, Croatia; ivan.borjan@kbsplit.hr (I.B.);; 2 School of Medicine, University of Split, Soltanska 2, 21000 Split, Croatia; 3 Department of Dermatology, University Hospital Split, Spinciceva 1, 21000 Split, Croatia; 4 Augenklinik Orasis, Titlisstrasse 44, 5734 Reinach, Switzerland; 5 Center for Eye Research and Innovative Diagnostics, Department of Ophthalmology, Institute for Clinical Medicine, Faculty of Medicine, Oslo University Hospital, University of Oslo, Kirkeveien 166, 0425 Oslo, Norway; 6 UKLONetwork, University St. Kliment Ohridski-Bitola, 7000 Bitola, North Macedonia

**Keywords:** type 1 diabetes mellitus, young-onset diabetes, proliferative diabetic retinopathy, risk factors, diabetic retinopathy screening, health economic model

## Abstract

*Background and Objectives:* To provide, for the first time, statistical data on risk factors for proliferative diabetic retinopathy (PDR) in young-onset type 1 diabetes (T1D) in Croatia, and to develop a predictive health economic model to evaluate the clinical and financial impact of implementing a national diabetic retinopathy (DR) screening. *Materials and Methods:* A cross-sectional study at University Hospital Split (June 2020–June 2022) analyzed 58 suitable T1D patients out of 562 screened. Patients were classified based on detailed fundus exams and photos into PDR and non-PDR groups. Clinical, demographic, and laboratory data were collected and analyzed using logistic regression. A health economic model was established to project the number of preventable PDR cases and the potential cost savings under various screening scenarios. *Results*: PDR was found in 47% of patients. Its presence was statistically significantly linked to longer diabetes duration, poor glycemic control, onset before 18 years of age, and irregular eye exams. Irregular ophthalmologic examinations increased the odds of PDR by nearly 30-fold. Health-economic modeling for 10,000 young-onset T1D patients showed that annual screening with 60% uptake could prevent about 1973 PDR cases and save €14.2 million annually. Screening remained cost-effective even with moderate uptake or less frequent intervals. *Conclusions*: Strict glycemic control and regular eye examinations are critical for preventing PDR in young T1D, and establishing a national screening program would be cost-effective, especially in resource-limited settings like Croatia, where providing appropriate, timely treatment may be challenging.

## 1. Introduction

Diabetic retinopathy (DR) is the most common cause of blindness among the working-age population worldwide [1]. This retinal microvasculopathy progresses in stages, beginning with non-proliferative abnormalities characterized by increased vascular permeability due to the breakdown of the blood–retinal barrier. Subsequently, it may advance to proliferative diabetic retinopathy (PDR) marked by severe ischemia and hypoxia leading to vascular proliferation, accompanied with or without fibrous tissue formation onto the retina, the optic nerve disc, and posterior surface of the vitreous. This progression can ultimately result in severe and irreversible vision loss [2]. Despite the potentially serious consequences, DR often remains undiagnosed until it progresses to more advanced stages. A recent pilot screening study for DR in Oslo, Norway, in newly diagnosed patients with diabetes mellitus (DM) showed that 62.8% had never had an eye exam [3].

According to the International Diabetes Federation (IDF), 177 million patients with DM globally have DR (around 30% of all), and approximately 41 million have PDR [1,2,4]. The number of patients with DR has been projected to rise to 224 million by 2045, with around 70 million of them experiencing vision-threatening DR [5]. It is important to highlight that individuals with type 1 diabetes (T1D) exhibit a notably higher prevalence of DR compared to those with type 2 diabetes (T2D). Studies reveal a prevalence of 77.3% for any DR and 32.4% for PDR in individuals with T1D, in contrast to 25.2% for any DR and 3.0% for PDR in those with T2D [6].

T1D accounts for about 5 to 10% of all DM cases worldwide [4,7,8]. Currently, almost 9.5 million people globally live with type 1 diabetes (T1D), and its incidence is steadily increasing by about 3–4% per year [4,7,8,9,10,11,12,13,14,15,16,17,18,19,20]. By 2040, it is predicted to increase to 13.5–17.4 million (60–107% higher than in 2021, when it was 8.4 million), with the largest relative increase in low- and middle-income countries [10].

Of note, 1.8 million children and adolescents below 20 years of age are estimated to have T1D, with around 222 thousand newly diagnosed cases annually [9]. An extensive epidemiological study across 17 European countries has shown a 3.9% annual increase in T1D incidence among the population aged 0–14 years [15]. Meanwhile, other studies revealed that the highest increase rates of 6.3% were observed in the youngest age group (0–4 years) [4,7,10,14,15,16,17,18,19,20]. However, strong geographic and national differences exist in this increasing trend [4,7,14,15,16,17,18]. For instance, the incidence rates range widely, from 0.1 per 100,000 children per year in Venezuela and China, to more than 50 per 100,000 per year in Finland [14,15,16,17,18,19,20,21,22]. The reason for this phenomenon remains unclear, but it is believed to be a result of an interplay between genetically predisposing factors and environmental triggers such as viral infections, toxins, some dietary factors or psychological stress [4].

According to the CroDiab registry, the prevalence of T1D among all patients with DM in Croatia in 2021 was 11.4% [23]. Meanwhile, in Croatian children and adolescents (0–14 years), the more recent annual increment of T1D is 5.87%, which is considerably lower than the 9% reported earlier (during the post-war period) but still significantly higher than the European average [19,20,24].

As the global prevalence of T1D continues to rise, individuals with T1D face an elevated risk of DR, including PDR. Although previous studies have shown that the most consistent risk factors for PDR are the duration of diabetes and poor metabolic control, significant individual differences have been observed, leaving the issue unanswered in specific countries [25,26,27,28]. The accurate number of T1D patients in the Croatian population affected by DR and PDR is unknown. However, severe and neglected cases of PDR are still frequently observed in routine retinological practice.

The aim of this study is to identify risk factors associated with the occurrence of PDR in young-onset T1D patients in the Croatian population. This is particularly noteworthy due to the high prevalence and a significant increase in the incidence of T1D in Croatian children and adolescents and a large number of cases with severe form of PDR with limited treatment possibilities. Given the substantial impact of PDR on the quality of life for patients with T1D, and its potential economic burden on the Croatian healthcare system, it is imperative to identify and address modifiable risk factors to prevent its occurrence and perform a predictive health economic model estimating the potential clinical and financial impact of implementing a DR screening program in Croatia.

## 2. Patients and Methods

The risk factors associated with the occurrence of PDR were analyzed in insulin-dependent (type 1), young-onset patients with diabetes (diagnosed before 30 years of age), examined and treated (if required) in the Retinal Section of the Eye Clinic, University Hospital Split, Croatia, during the period from June 2020 to June 2022.

This cross-sectional study was conducted following the Guidelines of the Helsinki Declaration, and it was approved by the Ethics Committee of the University Hospital Centre Split (Class: 500-03/21-01/129, Reference No.: 2181-147/01/06/M.S.-21-02). Signed informed consent was obtained from all participants included in the study.

**Figure 1:**
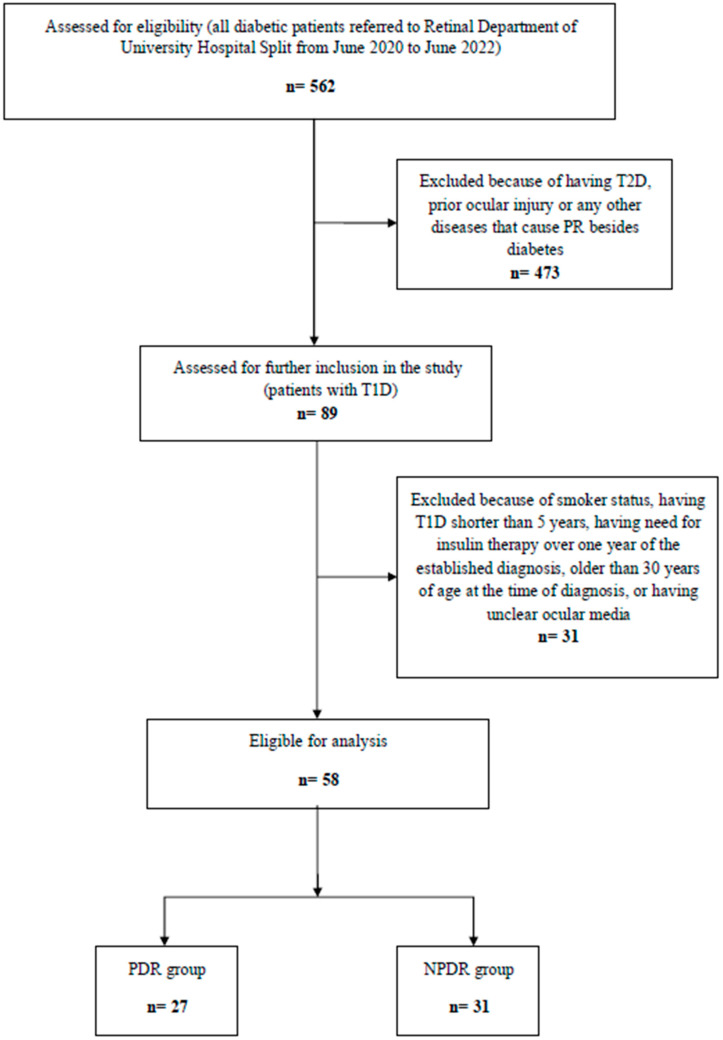
Flowchart of participant selection and group allocation. T2D = type 2 diabetes; T1D = type 1 diabetes; PR = proliferative retinopathy; PDR group = patients with proliferative diabetic retinopathy; NPDR group = patients without a sign of diabetic retinopathy or with nonproliferative diabetic retinopathy.

A total of 562 patients with DM were examined in the observed period. There were 89 patients with T1D, but only 58 met the inclusion criteria. For the inclusion: patients had to be younger than 30 years of age at diagnosis, required insulin therapy at the moment or at least within one year of the established diagnosis; the duration of DM in the patients had to be longer than five years; patients had to have clear ocular media to facilitate fundus examination; smokers were excluded due to well-known smoking impact on microcirculation [29,30]; patients with a history of ocular injury or any ocular disease (other than DM) that could have caused proliferative retinopathy were also excluded (Figure 1).

All patients underwent a detailed medical history evaluation. Data on all potentially relevant risk factors for PDR, including age at the time of diagnosis, diabetes duration, long-term glycaemic control, fundus findings and regularity of ophthalmological examinations and systemic hypertension history, were obtained from the patients’ medical records. Long-term average hemoglobin A1c (HbA1c) values equal to or less than 7% (53.0 mmol/mol) were considered as good long-term diabetes control, and all values above 7% (53.0 mmol/mol) were considered to be poor long-term control. The regularity of ophthalmologic examinations was estimated according to the international diabetes eye screening guidelines and was considered as regular or irregular [31].

Detailed ophthalmological examination, including fundus examination with an Ocular Mainster Wide Field lens following pupil dilatation by 1% tropicamide was performed in all patients by the same retinologist. Color fundus photographs were taken using Zeiss FF 450 plus IR fundus camera or Canon digital fundus camera (model: CX-1), and optical coherence tomography (OCT) (using Cirrus HD Model 400, Carl Zeiss, Oberkochen, Germany) was performed on all patients. The photographs and the OCT images were assessed by two independent, experienced retinologists. In some dubious cases (to confirm or exclude PDR), fluorescein angiography was performed. Each patient underwent a complete blood count and serum chemistry, including fasting blood glucose level, HbA1c, total cholesterol, high-density lipoprotein (HDL), low-density lipoprotein (LDL) and triglycerides.

The severity of DR was classified by the Early Treatment Diabetic Retinopathy Study (ETDRS) classification [32]. Classification of the patients was based on the fundus examination and color fundus photographs. In the case of bilateral affection, the patients were classified according to the findings in the worst affected eye with the most severe degree of retinopathy.

Patients were divided into two groups based on the assessed severity of DR. The first group (PDR group) was composed of 27 patients with evidence of PDR (neovascularisation on the retina surface and/or the optic nerve disc with or without vitreoretinal proliferation and vitreous hemorrhage). The second group (non-proliferative DR (NPDR) group) consisted of 31 diabetic patients without fundus changes consistent with DR, as well as patients with mild or severe NPDR characterized by microaneurysms, intraretinal hemorrhage, hard or cotton wool exudates, with or without macular edema, but with no presence of neovascularisation (Figure 1).

To assess the impact of age at the onset of T1D on the occurrence of PDR, each group of patients (PDR and NPDR) was divided into two subgroups: those with the onset of diabetes at or before 18 years of age, and those after the age of 18.

In order to assess the impact of diabetes duration on the occurrence of PDR, each group of patients (PDR and NPDR) was divided into two subgroups based on the median values of the duration of diabetes: those with a duration of diabetes of 22.5 years and below, and those above 22.5 years.

### 2.1. Health-Economy Model

A health-economic model was developed based on the data from the PDR prevalence and risk factors in young-onset T1D patients, as well as a pilot DR screening study conducted in the Oslo region, Norway, which reported low adherence to screening and significant associations between systemic risk factors and DR development [33]. Furthermore, a health-economic analysis compared a DR screening strategies including or not OCT in a Croatian DR screening cohort.

The data were extrapolated to a modeled population of 10,000 young-onset T1D patients in Croatia. For the purpose of economic modeling, we used a standardized hypothetical cohort of 10,000 young-onset T1D patients to allow proportional scaling and comparability; this number does not represent the exact current number of young-onset T1D individuals in Croatia. The model compared the expected number of PDR cases, screening costs, and potential cost savings from prevented cases across multiple implementation scenarios: (i) Screening frequencies: annual or biennial, (ii) Uptake levels: low (30%), medium (60%), high (90%), (iii) Cost inputs for combined screening (fundus + OCT)—fundus imaging (€40.16) and OCT examination (€36.71)—was set to €76.87 per patient per screening visit (assuming 4 visits/year on average); laser photocoagulation costs often required once per year (€84.32); intravitreal anti-vascular endothelial growth factor injections’ costs—on average 5 injections per year (€304.91) + costs of the drug (assuming use of aflibercept (€655.91); vitrectomy surgery in severe cases as day-surgery (€2011.55)—altogether accounting to €7207.45 per patient per year, (iv) Costs of procedures and drugs were obtained from the Croatian Institute of Health Insurance (https://hzzo.hr/poslovni-subjekti/hzzo-za-partnere/sifrarnici-hzzo-0 (accessed on 15 March 2025)). Preventive effect: 70% reduction in PDR cases with screening, based on logistic regression odds ratios from the Croatian study cohort.

### 2.2. Statistical Analysis

Qualitative data were presented in absolute and relative numbers. Quantitative variables were presented as arithmetic means ± standard deviation (SD) or medians and range, depending on the distribution. The distribution of quantitative variables was tested with the Kolmogorov–Smirnov test. The differences between the PDR and NPDR groups were tested by the Mann–Whitney U test if the distribution of quantitative variables deviated from non-normal distribution, and by *t*-test for normally distributed variables. The statistical significance of the differences in categorical characteristics was calculated by the chi-square test. The association between the occurrence of PDR and potential associated risk factors was assessed using logistic regression models. Statistical significance was set to *p* < 0.05, and all confidence intervals were given at a 95% level. The statistical package SPSS 24.0 (IBM Corp., Armonk, NY, USA) was used in the statistical data analysis.

## 3. Results

A total of 58 patients met the inclusion criteria for the study, including 27 (47%) patients from the PDR group and 31 (53%) patients from the NPDR group (Figure 1).

There were no statistically significant differences between patients in the observed groups by age (t = 0.505; *p* = 0.616) and sex (χ^2^ = 0.04; *p* = 0.849). In the PDR group, there were 12 (44%) females and 15 (56%) males, while in the NPDR group, there were 13 (42%) females and 18 (58%) males. The median age in the PDR group was 49 years (min-max: 28–58 years), while in the NPDR group was 47 years (min–max: 35–55 years) (Table 1).

The median diabetes duration was 11 years higher (95% CI: 5.5–16.5) in the PDR group than in the NPDR group (Z = 4; *p* < 0.001). Additionally, the median age at onset of diabetes was 10 years lower (95% CI: 6.2–13.8) in the PDR group (Z = 4.5; *p* < 0.001).

The mean value of systolic blood pressure (BP) in the PDR group was 10 mmHg higher than in the NPDR group (t = 3.58; *p* = 0.001). The PDR group also had significantly higher current fasting blood glucose levels, current HbA1c values (at the time of examination), as well as diastolic BP, LDL and triglycerides values (Table 1). However, there was no statistically significant difference in the total cholesterol levels between the two observed groups (t = 0.784; *p* = 0.436).

The long-term serum glucose control was also compared between the groups. In the PDR group, 23 (85%) out of 27 patients had poor long-term blood glucose control, in contrast to only 9 patients (29%) in the NPDR group (χ^2^ = 16.2; *p* < 0.001) (Table 1). Long-term HbA1c levels higher than 7% (53.0 mmol/mol) increased the odds for PDR by 14 times [OR = 14 (95% CI: 3.8–52); *p* < 0.001] (Table 2).

Similar differences between the groups were observed comparing the data on the regularity of ophthalmological examinations (χ^2^ = 24.7; *p* < 0.001) (Table 1). Only 5 (19%) of the 27 patients in the PDR group had regular ophthalmological check-ups, compared to 27 (87%) of the 31 patients in the NPDR group. The irregular ophthalmologic examinations increase the odds for having PDR by 29.7 times [OR = 29.7 (95% CI: 7.1–124); *p* < 0.001] (Table 2).

The onset of diabetes at or before 18 years of age was more common in the PDR group. In the NPDR group, only 4 (13%) patients with DM were diagnosed before the age of 18, compared to 16 (59%) patients in the PDR group (χ^2^ = 11.7; *p* = 0.001). Patients with onset of DM at or before 18 years of age had 9.8 times higher odds of developing PDR compared to those who developed the disease after the age of 18 [OR = 9.8 (95% CI: 2.7–36); *p* < 0.001] (Table 2).

When the patients in the PDR and NPDR groups were divided into two subgroups based upon the median values of the duration of diabetes (below and above 22.5 years), only 7 (25.9%) out of 27 patients in the PDR group had a duration of diabetes of 22.5 years or less, compared to 22 (71%) out of 31 patients in the NPDR group (χ^2^ = 9.9; *p* = 0.002). Having a duration of diabetes above 22.5 years increased the odds for having PDR 7 times [OR = 7 (95% CI: 2–22.2); *p* < 0.001] (Table 2).

Considering a 60% annual screening uptake, 6000 patients would be screened in Croatia annually at a cost of €0.46 million (Table 3). Without screening, an estimated 2820 patients (47% of 6000) would develop PDR over time. With screening, approximately 1973 cases of PDR could be prevented, leading to cost savings of €14.2 million in avoided treatment.

Across scenarios, the net economic impact ranged from €7.1 million to over €21 million in annual savings in Croatia, depending on the uptake and screening frequency. Even with biennial screening, medium uptake yielded an estimated €14.2 million in net savings (Table 3).

The inclusion of OCT in all exams, as shown in the Norwegian model, can reduce the need for return visits, and further enhance cost-effectiveness by minimizing patient transport and time costs (Table 3).

## 4. Discussion

Our study showed a 47% prevalence of PDR in the young-onset T1D patients having diabetes duration between 10 and 40 years. The result is consistent with previous studies that have shown a PDR prevalence ranging from 13% to 50% depending on the T1D duration [34,35,36]. The Wisconsin Epidemiologic Study of Diabetic Retinopathy (WESDR) reported a PDR prevalence of even 67%, after 35 years or more of having diabetes [34,35,37]. In accordance with the higher occurrence of PDR with a longer duration of T1D, our study showed 11 years longer duration of diabetes in the PDR group. Our results differ from recent studies that reported a significantly lower prevalence of PDR after 12–28 years of T1D duration (Surowiec et al. 8.1%; Heintz et al. 8.5%) [25,38].

Another risk factor associated with the more frequent development of PDR was the age of onset of T1D. In the PDR group, patients developed DM 10 years earlier than those in the NPDR group. The odds for PDR were 9.8 times higher in patients with DM onset at/or before 18 years of age, compared to those who developed the disease after the age of 18. The proportion of patients with onset of DM at/or before 18 years of age was 4.5 times higher in the PDR group than in the NPDR group. There appears to be a considerable disagreement in the literature in regard to the influence of the latter risk factor on the progression of retinopathy. While Laiginhas et al. found a similar risk of PDR occurrence in the early and late-onset T1D group, many other reports have suggested age at the T1D diagnosis may have an important role in the PDR prevalence [26,36,37,39]. As noted by Jia et al., although patients diagnosed at a young age have a longer time interval being free of PDR regardless of the duration of diabetes (progression of retinopathy is almost non-existent in young-onset DM at <13 years of age), they are at a higher risk of developing PDR compared to those diagnosed in adulthood [40]. This may be attributed to the fact that an earlier age at DM onset means a longer exposure to metabolic disorders. During puberty, hormonal changes and a decrease in insulin sensitivity can exacerbate the metabolic control of DM [41]. These changes, along with physical and psychological maturation, which reduce therapeutic compliance, can increase the risk for developing PDR. In contrast, other sources suggest that although hormonal changes and puberty contribute to the progression of diabetic microvascular diseases, sex hormones in puberty might offer some protection against PDR [40,41].

Poor long-standing glycaemic control, as well as late and/or irregular ophthalmological examinations, were found to be also important risk factors associated with PDR in our study. The proportion of patients with poor long-term blood glucose control was 2.9 times higher in the PDR group compared to the NPDR group, which aligns with previous findings published [11,16,25,39,42]. The U.K. Prospective Diabetes Study (UKPDS) showed that for each percentage point decrease in HbA1c, there is a 35% reduction in the risk for DR and 15% for PDR [42]. Lind et al. found a 1% (10 mmol/mol) increase in HbA1c level resulted in an odds ratio of 2.87 for PDR [43]. Additionally, in the PDR group, our study found 6.2 times more patients with irregular ophthalmological examination than in the NPDR group. While numerous studies emphasize the significance of regular ophthalmological examinations for T1D patients and provide guidelines for their frequency [31,43,44,45], to our knowledge, this study is the first to compare the occurrence of PDR between individuals who regularly attended their appointments and those who did not.

Our results found no association between the occurrence of PDR and sex, which is consistent with findings from other studies [25]. However, Jansson et al. found the male gender to be a significant predictor of DR severity [27].

Hypertension was associated with PDR in our study, as well as in previous research [25,28,42]. In contrast, Klein and co-authors found that blood pressure was not a predictor of the incidence or progression of PDR [46]. Our study also confirmed the findings of the previous studies, which reported significantly higher levels of LDL and triglycerides in patients with PDR [47,48]. No significant association was found between total serum cholesterol levels and PDR, which is consistent with the UKPDS [42].

Based on our findings, and the fact that certain risk factors for PDR cannot be modified (duration and onset of T1D, genetic predisposition, stress), it is crucial to prevent PDR by improving glucose control (using personalized models of glucose control and intensive treatment) and by diagnosing DM complications through regular screenings when treatment is still possible [31,49,50]. Recent studies suggest that screening and regular monitoring programs for DR are not only effective but also economically viable [3,33,51,52]. Unfortunately, in Croatia, there are no currently established national recommendations or clinical guidelines for DR screening and treatment regardless of T1D [53]. Therefore, our focus should be on the prevention and earlier detection of DR, achieved through better patient education and well-designed, cost-effective national screening program with regular ophthalmological follow-up and timely management. It is recommended that the first fundus examination in T1D patients should be within 3 to 5 years after the diagnosis of DM or at puberty, whichever is earlier. If there are no signs of DR, the examination should be repeated yearly. Once retinopathy is detected, the frequency of assessments may need to increase depending on its severity. This allows timely treatment and prevents progression to PDR with serious consequences (often bilateral blindness) [2,31,44,45]. Thus, it is necessary to emphasize good interdisciplinary cooperation between ophthalmologists, diabetologists and pediatricians.

This health economic analysis underscores the substantial potential for cost savings and clinical benefit from implementing a systematic DR screening program in Croatia, particularly for young-onset T1D patients at high risk of PDR. The results are consistent with the Norwegian findings, where a combined imaging strategy not only improved diagnostic yield but was also economically superior [33,54,55,56]. Our Croatian cohort data demonstrates the devastating impact of irregular eye exams, with nearly 30-fold increased risk of PDR. Our predictive model suggests that even modest screening uptake (60%) with annual imaging could prevent nearly 2000 cases of PDR and generate multimillion-euros healthcare savings. These findings support the urgent need for a national DR screening initiative in Croatia, with structured pathways for regular follow-up, especially in resource-limited areas. Investment in infrastructure and teleophthalmology, alongside combined OCT-fundus exams, should be prioritized as cost-saving interventions that preserve vision and quality of life [57].

The main limitation of this study is the relatively small sample size. However, that was the total number of T1D patients referred to our clinic during the specified period who met the strict inclusion criteria for the study, and we believe that this limitation should not affect our results significantly.

## 5. Conclusions

Rigorous glycemic control and regular ophthalmological examinations are key to preventing PDR in early-onset T1D. Our findings support the urgent need for a national DR screening program in Croatia, particularly given the high incidence of T1D in Croatian youth and the frequent occurrence of severe PDR in these patients. Implementing systematic screening would allow earlier identification of at-risk individuals and could prevent up to two-thirds of PDR cases, resulting in significant savings for the healthcare system (between €7 and €21 million annually), which is especially important in settings with limited resources where timely treatment may be challenging. Addressing modifiable risk factors, early detection through a structured, national screening program and prompt treatment of DR substantially reduce the clinical and economic burden of PDR. Establishing such a program would prevent vision loss in hundreds of individuals each year and enable long-term savings and sustainability in healthcare, so the implementation of national screening should be a priority.

## Figures and Tables

**Table 1:** Comparison of demographic characteristics and laboratory findingsbetween two groups of young-onset patients with diabetes according to the retinopathy status (with or without proliferative diabetic retinopathy).

Variable	PDR (*n* = 27)	NPDR (*n* = 31)	*p*
Age (years)			0.616 *
Median [min–max]	49 [28–58]	47 [35–55]	
Sex *n* (%)			0.849 ^‡^
Female	12 (44)	13 (42)	
Male	15 (56)	18 (58)	
Duration of diabetes (years)			<0.001 *
Median [min–max]	31 [15–40]	20 [10–27]	
Age at onset of diabetes (years)			<0.001 *
Median [min–max]	18 [10–30]	28 [17–30]	
Current glycosylated hemoglobin (HBA1c) level (%)			<0.001 ^†^
(mean ± SD)	7.9 ± 1.4	5.9 ± 0.7	
Systolic BP (mm Hg)			0.001 ^†^
(mean ± SD)	139 ± 10	129 ± 10	
Diastolic BP (mm Hg)			0.014 ^†^
(mean ± SD)	86 ± 4.7	81 ± 9.2	
Current blood glucose level (mmol/L)			<0.001 ^†^
(mean ± SD)	7.5 ± 1.4	6.0 ± 0.7	
Total cholesterol (mmol/L)			0.436 ^†^
(mean ± SD)	4.34 ± 1.10	4.14 ± 0.81	
LDL (mmol/L)			<0.001 *
Median [min–max]	2.6 [1–4]	1.7 [1–3]	
Triglycerides (mmol/L)			0.001 *
Median [min–max]	1.9 [0.9–2.7]	1.5 [0–2]	
Long-term blood glucose control ^§^ *n* (%)			<0.001 ^‡^
Good (HbA1c ≤ 7%)	4 (15)	22 (71)	
Poor (HbA1c > 7%)	23 (85)	9 (29)	
Regularity of ophthalmological examinations ^‖^ *n* (%)			<0.001 ^‡^
Regular	5 (19)	27 (87)	
Irregular	22(81)	4 (13)	

* Mann–Whitney U test; ^†^ *t*-test; ^‡^ chi-square test. Values are presented as median (min–max), as mean (±SD) or as number (%). PDR group = patients with proliferative diabetic retinopathy; NPDR group = patients without a sign of diabetic retinopathy or with non-proliferative diabetic retinopathy; SD = standard deviation; BP = blood pressure; LDL = low-density lipoprotein. ^§^ Based on the long-term values of glycosylated hemoglobin (HbA1c), data obtained from patients’ medical records ^‖^ According to the International diabetic retinopathy screening guideline, or according to the recommendation of the competent ophthalmologist (data from medical history records).

**Table 2:** Univariate logistic regression analysis results assessing variables associated with the occurrence of proliferative diabetic retinopathy.

Variable	PDR *n* (%)	NPDR *n* (%)	p *	OR (95% CI)	*p* ^†^
Long-term blood glucose control ^‡^					
Good (HbA1c ≤ 7%) ^‖^	4 (15)	22 (71)	<0.001	14 (3.8–52)	<0.001
Poor (HbA1c > 7%)	23 (85)	9 (29)			
Regularity of ophthalmological examinations ^§^					
Regular ^‖^	5 (19)	27 (87)	<0.001	29.7 (7–124)	<0.001
Irregular	22 (81)	4 (13)			
Age of onset of diabetes (years)					
≤18	16 (59)	4 (13)	0.001	9.8 (2.7–36)	<0.001
>18 ^‖^	11 (41)	27 (87)			
Duration of diabetes (years)					
≤22.5 ^‖^	7 (26)	22 (71)	0.002	7 (2–22.2)	<0.001
>22.5	20 (74)	9 (29)			

* chi square test; ^†^ logistic regression ^‖^ Reference level. Values are presented as number (%). PDR group = patients with evidence of proliferative diabetic retinopathy; NPDR group = patients without a sign of diabetic retinopathy or with non-proliferative diabetic retinopathy. ^‡^ Based on the long-term values of glycosylated hemoglobin (HbA1c), data obtained from patients’ medical records. ^§^ According to the International diabetic retinopathy screening guideline or according to the recommendation of the competent ophthalmologist (data from medical history records).

**Table 3:** Annual cost savings and proliferative diabetic retinopathy cases prevented based on different screening frequencies and uptake levels in a modeled Croatian T1D population.

Scenario	Population Screened	Screening Cost (Euros/€)	Expected PDR Cases w/o Screening	PDR Cases Prevented	Net Economic Impact *
Annual—Low Uptake (30%)	3000	€230,610.0	1410	987	€7,113,753.24
Annual—Medium (60%)	6000	€461,220.0	2820	1973	€14,220,298.85
Annual—High (90%)	9000	€691,830.0	4230	2961	€21,341,259.45
Biennial—Medium (60%)	6000 (every 2 years)	€230,610.0	2820	~1973	€14,220,298.85

* Net Economic Impact = PDR Cases Prevented × Screening Cost Per Patient (€76.87) × Cost for Combined Screening (€5060.65).

## Data Availability

The datasets used and/or analyzed during the current study available from the corresponding author on reasonable request.

## List of Abbreviations

DR, Diabetic retinopathy; PDR, proliferative diabetic retinopathy; IDF, International Diabetes Federation; T1D, Type 1 diabetes; OCT, optical coherence tomography; HDL, high-density lipoprotein; LDL, low-density lipoprotein; HbA1c, hemoglobin A1c; ETDRS, Early Treatment Diabetic Retinopathy Study; SD, standard deviation; WESDR, Wisconsin Epidemiologic Study of Diabetic Retinopathy; UKPDS, The U.K. Prospective Diabetes Study.
